# Green methods of lignocellulose pretreatment for biorefinery development

**DOI:** 10.1007/s00253-016-7884-y

**Published:** 2016-10-06

**Authors:** Laura Capolupo, Vincenza Faraco

**Affiliations:** 1 Department of Chemical Sciences, University of Naples “Federico II”, Complesso Universitario Monte S. Angelo, via Cintia, 4, 80126 Naples, Italy; 2 European Center “Europe Direct LUP”, Complesso Universitario Monte S. Angelo, via Cintia, 4, 80126 Naples, Italy; 3 Interdepartmental Center “R. d’Ambrosio, LUPT”, Complesso Universitario Monte S. Angelo, via Cintia, 4, 80126 Naples, Italy

**Keywords:** Biorefinery, Lignocellulose, Extrusion, Steam-explosion, Liquid Hot Water, Ammonia Fiber Explosion, Supercritical CO_2_ explosion, Organosolv, Ozonolysis, Ionic liquids, Biological pretreatment.

## Abstract

Lignocellulosic biomass is the most abundant, low-cost, bio-renewable resource that holds enormous importance as alternative source for production of biofuels and other biochemicals that can be utilized as building blocks for production of new materials. Enzymatic hydrolysis is an essential step involved in the bioconversion of lignocellulose to produce fermentable monosaccharides. However, to allow the enzymatic hydrolysis, a pretreatment step is needed in order to remove the lignin barrier and break down the crystalline structure of cellulose. The present manuscript is dedicated to reviewing the most commonly applied “green” pretreatment processes used in bioconversion of lignocellulosic biomasses within the “biorefinery” concept. In this frame, the effects of different pretreatment methods on lignocellulosic biomass are described along with an in-depth discussion on the benefits and drawbacks of each method, including generation of potentially inhibitory compounds for enzymatic hydrolysis, effect on cellulose digestibility, and generation of compounds toxic for the environment, and energy and economic demand.

## Introduction

Biomasses have been considered, in the last years, a renewable and sustainable source of organic carbon with zero carbon emission (Zhou et al. 2011; Long et al. 2013) and seem to be the most promising resources to produce green products and replace fossil resources that are limited and show many hurdles such as greenhouse gas emissions and elevated prices.

Among plant-based raw materials, lignocellulosic biomasses are the most abundant, low-cost, bio-renewable biomasses (Balan 2014) that do not interfere with food supply and derive in large quantities from forestry, agricultural, and agroindustrial wastes (Limayem and Ricke 2012; Saini et al. 2015). For this reason, lignocellulosic biomasses have great importance as the prime source of biofuels and other bioproducts (such as succinic acid or lactic acid) that can be utilized as building blocks for production of new polymers (Isikgor and Becer 2015; Adsul et al. 2011).

Over the next few years, in order to minimize the dependence on fossil-based resources and petroleum industry and thanks to new research trends in the production of green chemicals from different sources, the second generation biorefineries continue to gain more importance (Fahd et al. 2012; Cherubini 2010). The biorefinery can be defined as the conversion of biomasses into a wide range of biomaterials and bioenergy (biofuels, heat, etc.). Moreover, this exponential growth of biorefinery concept contributes to the development of circular economy model (Anwar et al. 2014; Liguori and Faraco 2016). In circular economy model, lignocellulosic materials, used to generate bio-based products, are recovered and recycled and used again and again (Velis and Vrancken 2015). Therefore, this economic model represents an alternative to the linear economic model (extraction–production–use–waste disposal), used in the past in petroleum industries.

Obviously, during the development of biorefineries, moving from petroleum to biomasses as the carbon feedstock, it is necessary to be sure that the environment is protected. For this reason, the integration of “green chemistry” is mandatory (Clark et al. 2009). In particular, the overall purpose of “green chemistry” is to develop new technologies that aim at eliminating the use and/or generation of environmentally hazardous chemicals and at producing “green” and sustainable chemical products with minimized energy demands, through the use of sustainable feedstocks. Therefore, the final products of “green biorefineries” should be non-toxic, environmentally benign, should degrade into safe chemicals, and it should be recyclable or at least produce not many wastes.

The general scheme for lignocellulosic bioconversion in biorefinery approach involves the following steps: pretreatment step to deconstruct cell walls into its components, an eco-friendly enzymatic hydrolysis, fermentation of monosaccharides derived from previous step into target products, separation of residues, and finally, purification of the products. Obviously, each step has its difficulties that add costs to the overall process.

The pretreatment step is the most costly in the whole process but it is crucial for enhancing enzymes accessibility during saccharification step, exposing the cellulose, breaking down the lignin structure, altering both its structural and chemical properties, and break down the crystallinity of cellulose.

At the same time, pretreatment should prevent destruction of cellulose and hemicellulose, generation of inhibitors for subsequent steps and the degradation or loss of sugars; it should minimize energy demand and costs, chemical reactive consumption and improve the formation of sugar monomers in hydrolysis step (Adsul et al. 2011).

The enzymatic hydrolysis step is another costly step due to the price of the enzymes, and it is hard because polysaccharide components are hindered by many physico-chemical, compositional, and structural factors, such as lignin that cover them (Gupta et al. 2011).

Efficient fermentation step also is crucial for the development of biorefinery; in fact, monosaccharides derived from saccharification step, can be converted into bioproducts that can be building blocks for production of new polymers. Instead, the overall cost of fermentation step depends on the type of starting biomass.

This review is focused on pretreatment methods because this step has a great potential to improve the efficiency of the overall biorefinery process, although it is usually an expensive step with respect to energy (Mosier et al. 2005). In particular, the objective is to deepen “green” pretreatment processes for the production of bioenergy (such as biofuels or heat) and biomaterials. In particular, the benefits, the drawbacks and the effects of each pretreatment method on the biomass, considering the process in terms of generation of compounds potentially inhibitory for enzymatic hydrolysis or toxic for the environment, recycle of chemicals and energy and economic demand are summarized.

### Pretreatment methods for lignocellulosic biomass

Many types of lignocellulosic biomass pretreatment were developed and they can be classified into four different categories: physico-chemical, physical, chemical and biological (Mood et al. 2013; Kumar and Wyman 2009). Table 1 summarizes different pretreatment methods for different feedstock, with their pros and cons and the yields of monosaccharides obtained after hydrolysis. Because there is a wide variety of feedstock, each one with different characteristics, a single method cannot represent the universal choice for all types of lignocellulosic biomass. Ongoing research is focusing on optimizing, simplifying and improving “green” pretreatment technologies in order to reduce energy demands, environmental impact, the use of chemicals and catalysts, formation of by-products and wastes, to develop an economically feasible biorefinery and at the same time to improve lignocellulose’s digestibility. In fact, some already developed methods employ the use of harsh chemicals and severe conditions that lead to waste treatment problem with a consequent increase of environmental pollution and costs. Moreover, some of these methods have a negative effect on the efficiency of the enzymes used during saccharification step and sometimes they can produce by-products that can inhibit the growth of microorganisms during fermentation step. Therefore, usually extra costs are necessary to solve the negative effect of pretreatment on subsequent steps. For these reasons, in this review, pretreatment processes that are nowadays considered as “green” methods are deepened.

**Table 1:** Summary of various pretreatment methods for different feedstock, their advantages and disadvantages, sugar percent yields, and selected references

*Pretreatment methods*	*Feedstock*	*Sugar yield*	*Advantages*	*Disadvantages*	*Selected references*
Extrusion	• Agricultural residuals: rice straw, corn stover, and wheat straw	50–75 % Reducing sugar	• Moderate temperature • Good sugar yields • Adaptability for many process modifications • Less hazardous process	• Partially hemicellulose degradation • Generation of inhibitor compounds • Incomplete destruction of lignin-carbohydrate matrix	• Lamsal et al. 2010 • Yoo 2011b
Steam-explosion	• Agricultural residuals: wheat straw, corn stalk, and sugarcane • MSW • Hardwood •Forest residuals	50–70 % Reducing sugars	• Good sugar recovery • Cost-effective • Less hazardous process • Lignin transformation and hemicellulose solubilization	• Partially hemicellulose degradation • Generation of inhibitor compounds • Incomplete destruction of lignin-carbohydrate matrix	• Ruiz et al. 2008 • Sharma et al. 2015
Liquid hot water (LHW)	• Agricultural residuals: sugarcane bagasse, corn stover, wheat straw, and sunflower stalks • MSW	80–94 % Reducing sugars	• Recovery of almost pure hemicellulose • No addition of catalyst or chemicals • Hydrolysis of hemicellulose • No need for size reduction • High sugar recovery • Little formation of inhibitors	• High energy demand • Solid mass left over will need to be dealt with	• Rogalinski et al. 2008 • Perez et al. 2008
Ammonia fiber explosion (AFEX)	• Agricultural residuals: wheat straw, corn stover, bagasse, and rice straw • Municipal solid wastes	Up to 80–90 % of reducing sugars	• Efficient removal of lignin • Low formation of inhibitor compounds and formation of sugars degradation • Moderate process conditions	• Costly process • No efficient for biomass with high lignin content	• Balan et al. 2009 • Bradshaw et al. 2007
Supercritical CO_2_ explosion	• Agricultural residuals: wheat straw, sugarcane bagasse	Up to 90 % of reducing sugars	• Increases accessible surface area • Do not imply generation of toxic compounds	• Costly process • Very high pressure requirements	• Zheng et al. 1995 • Zhang and Wu 2014
Organosolv	• Agricultural residuals: wheat straw, sugarcane bagasse	Up to 60 % of reducing sugars	• Causes lignin and hemicellulose hydrolysis • Pure lignin removal as byproduct	• Costly process • Solvents need to be drained and recycled	• Mesa et al. 2011 • Zhao et al. 2009a
Ozonolysis	• Agricultural residuals: wheat straw, bagasse, and peanut and poplar sawdust	45–90 % Reducing sugars	• Efficiently degradation of lignin • Does not imply generation of toxic compounds • Moderate reaction conditions	• Costly process • Large amount of ozone necessary	• Garcia-Cubero et al. 2009 • Travaini et al. 2013
Ionic liquids (ILs)	• Agricultural residuals: wheat straw, bagasse, peanut and poplar sawdust, and corn stover	60–85 % Reducing sugars	• Efficiently dissolution of cellulose	• Great amounts of expensive ILs are needed • Solutions viscous and difficult to handle	• Liu et al. 2012 • Qiu et al. 2012
Biological	• Agricultural residuals: wheat straw, rice straw • Softwood	20–50 % Reducing sugars	• Low energy imput • Moderate reaction conditions • No addition of catalyst or chemicals • Do not imply generation of toxic compounds • Cost-effective	• Low rate of hydrolysis • Necessity of a large sterile area	• Wan and Li 2012 • Sánchez 2009

### Physical and physico-chemical pretreatment

Many types of physical and physico-chemical pretreatment were developed, each one with its advantages and disadvantages. Between these methods, extrusion, liquid hot water, steam-explosion, ammonia fiber explosion, and supercritical CO_2_ explosion are the most effective and environmentally friendly available processes. They were optimized for a wide variety of feedstock and have been tested on pilot scale for industrial applications.

The disadvantage of using harsh conditions, typical of these methods, is balanced with the advantage of obtaining high sugar yields, without addition of chemicals or addition of non-toxic chemicals that can be recycled, reducing operating costs.

#### Extrusion pretreatment

Extrusion is an innovative and promising continuous physical pretreatment process that exposes cellulose, preserves the hemicellulose and avoids the inhibitory compounds formation and degradation of sugars, using inexpensive chemicals and simple and cost-effective equipment. In this pretreatment, materials are introduced into the extruder and then transported along the length of the barrel with a driving screw (Lamsal et al. 2010). During the process, biomasses undergo mixing, heating, and shearing upon pressure release at the finishing end (similar to steam-explosion pretreatment). This results into physical and chemical alteration of cellulose, hemicellulose, and lignin, leading to increased enzymatic accessibility of cellulose (Yoo et al. 2011a) and a consequent enhancement of sugar recovery that was demonstrated due to increase in surface area (Karunanithy and Muthukumarappan 2011a; Karunanithy and Muthukumarappan 2011b) and pore size (Jurisic et al. 2009). A wide variety of lignocellulosic biomasses, such as rice straw (Chen et al. 2011), switchgrass (Karunanithy and Muthukumarappan 2010a), corn stover (Zhang et al. 2012a), and wheat straw (Vandenbossche et al. 2014), have been pretreated with this method. Parameters influencing extrusion pretreatment include reaction time, pressure, biomass dry matter, and particle size. Moreover, modifying the bioreactor parameters, such as barrel temperature and screw configuration and speed, it is possible to attain an optimized process with high efficiency.

For example, Karunanithy and Muthukumarappan (2010b) reported that sugar recovery from corn stover changed by modifying extrusion conditions, such as screw speed (from 25 to 125 rpm) and barrel temperature (from 25 to 125 °C) and enzymes used during the hydrolysis step. Their study showed that the highest glucose and xylose yields (75 and 49 %, respectively), 1.96 and 2.0 times higher than the untreated corn stover, were recorded at screw speed of 75 rpm and highest temperature, with the addition of β-glucosidase and cellulase (1:4 ratio).

Moreover, depending on the intensity of screw stress, it can be observed also thermal degradation of sugars. Moreover it is important to mention that extrusion pretreatment can be combined with other methods to improve the less sugar recovery observed using extrusion method alone.

For example, Zhang et al. (2012a, 2012b) evaluated the effect of extrusion and alkali-assisted extrusion on pretreated corn stover digestibility. They showed that in extrusion pretreatment, the optimum glucose and xylose yields were 48.79 and 24.98 %, respectively, that are 3.3 and 13.3 higher than untreated materials. On the other hand, in combined process (alkali loading of 0.04 g/g dry biomass), glucose and xylose yields were 86.8 and 50.5 %, respectively, 2.2 and 6.6 times greater than their untreated counterparts. Probably this is due to greater delignification due to the combination of extrusion and alkali process.

In order to develop extrusion pretreatment at industrial scale an economic analysis of the extrusion pretreatment methods should be evaluated. For example, Yoo (2011b) analyzed costs of extrusion pretreatment in comparison to dilute acid pretreatment for lignocellulosic ethanol production over a year of production, using soybean hull as raw material. The production cost of each pretreatment was evaluated at the plant scale and the results showed that the total fixed capital investment for each pretreatment was 174 and 191 million, respectively, and the total pretreatment equipment costs for extrusion and dilute acid hydrolysis were estimated at about 25 and 27 million, respectively. These results demonstrate that extrusion pretreatment is a promising pretreatment technology compared to dilute acid hydrolysis due to lower capital and pretreatment cost because of simpler and less equipment are necessary.

#### Advantages and drawbacks

The main advantages of the extrusion method are short residence time, rapid mixing, no washing step, possibility of continuous operation, and consequently applicability for easy scale-up (in fact, extruder systems are already commercially available; for example, this type of systems were developed by Lehmann Maschinenbau company) and moderate temperature (Karunanithy and Muthukumarappan 2011c). These moderate temperatures employed in the process and conditions prevent formation of fermentation inhibitors and decrease monomeric sugars degradation (Yoo et al. 2011a; Lee et al. 2010). Further, it has no solid loss, no fermentation inhibitors formation, unnecessary washing step and no requirements for important safety issues. Moreover, an important pro is the adaptability of the process for many different process modifications (such as high pressure, acid, or alkali addition) (Vandenbossche et al. 2014). This is considered also a con due to the less sugar recovery obtained with this method after enzymatic hydrolysis, respect to others.

#### Steam-explosion (SE)

Steam-explosion (SE) is the most commonly adopted physico-chemical pretreatment method for lignocellulosic biomasses. It can be performed with or without addition of chemicals (catalyzed SE or SE, respectively). SE breaks down structural components of biomass by steam-heating, shearing, and auto-hydrolysis of glycosidic bonds of hemicellulose. Originally, SE was a batch technology, but many efforts have been carried out to perform this pretreatment in a continuous way in order to obtain a better control of the operation variables, better heat transfer, and lower accumulation of by-products. In this process, biomass materials are subjected to high temperatures (at 160–260 °C) and pressures (20–50 bar) for a few seconds (30 s) to several minutes (20 min), and then the pressure is released to the atmospheric one, causing an explosion (Varga et al. 2004; Glasser and Wright 1998). At the end of the process, a material called slurry can be recovered that contains a cellulose-rich fraction, a hydrolyzed hemicellulose fraction and lignin-rich filtrate that can be removed from the material are obtained. (Wang et al. 2010; Sun et al. 2005). This leads to increased accessible surface area, enzyme accessibility, hydrolysis rate, and cellulose digestibility (Kabel et al. 2007; Duff and Murray 1996). For example, Viola et al. (2008) reported that SE pretreatment of wheat straw improved the digestibility by 25 % compared to untreated wheat straw.

The parameters affecting SE efficiency are particle size, residence time, and temperature (Brownell et al. 1986; Duff and Murray 1996). In particular, increase in temperature might increase removal of hemicellulose, but it increases sugars degradation, resulting in a decrease in total sugar yields (Ruiz et al. 2008).

SE is actually considered one of the most efficient pretreatment processes to treat many kinds of biomasses, including hardwood (Estevez et al. 2012), agricultural (Ballesteros et al. 2002) and municipal wastes (Dereix et al. 2006), and forest residuals (Hooper and Li 1996). As a matter of fact, it has been developed at pilot scale (Sharma et al. 2015). Two of the most famous industrial scales SE are held by Iogen Corporation in Canada (Zheng et al. 2009) and by Masonite batch process (Mosier et al. 2005).

SE process was simulated in order to study the economic feasibility of the process for biogas production from wheat straw. Total project investment for a biogas production plant, with 200,000 tons/year of wheat straw as feedstocks, was calculated to be 61.8 million euros. On the other hand, the total project costs for a plant without a SE pretreatment unit was lower: 56.4 million euros. However, the average annual production of biogas from wheat straw increase from 36.8 to 70.5 million Nm3/year if pretreatment unit is used (Shafiei et al. 2013).

The other type of SE pretreatment, catalyzed SE, addition of an acid, such as H2SO4, can decrease retention time and temperature, improve hydrolysis rate, decrease the production of inhibitors and lead to an almost complete removal of hemicellulose and lignin (Ballesteros et al. 2002; Stenberg et al. 1998). Moreover, several detoxification methods have been studied in order to reduce the inhibitory effect, but it is necessary to avoid these problems. Total project investment for the construction of a 83 million liters per years plant, using sugarcane bagasse as feedstocks, was calculated to be 21.9 million dollars (Gubicza et al. 2016).

#### Advantages and drawbacks

The main advantages of this method include low environmental impact and costs, high sugar yields (Cara et al. 2006; Ballesteros et al. 2002), less hazardous processes, small energy demand, unnecessary recycling, and unnecessary addition of chemicals (Avellar and Glasser 1998). In particular, the cost of the overall process is greatly reduced because different particle size can be utilized. In fact, obtaining small chip sizes represents a third of overall energy cost of the process (Wooley et al. 1999).

Moreover, hydrolysis step is facilitated by water that acts as an acid at high temperature (Weil et al. 1997) and by organic acids, such as acetic acid, generated at high temperature from auto-hydrolysis of acetyl or other functional groups associated with hemicellulose.

At the same time, acidic conditions and high temperatures are the main drawbacks of SE. In fact, with these conditions, the partial hemicellulose degradation and the generation of some toxic compounds that could influence enzymatic hydrolysis and fermentation step are observed (Cantarella et al. 2004). The major inhibitors are furan derivatives, weak acids, and phenolic compounds. The furan derivatives such as furfural and HMF (5-hydroxymethylfurfural) have been reported as inhibitor by prolongation of the lag phase during batch fermentation (Palmqvist and Hahn-Hägerdal 2000). Weak acids, such as acetic, formic, and levulinic acid, can inhibit cell growth due to the inflow of undissociated acid into the cytosol (Palmqvist and Hahn-Hägerdal 2000). Finally, phenolic compounds, derived from lignin breakdown, have been suggested to inhibit the fermentation of lignocellulosic hydrolysates. However, the mechanism of the inhibiting effect has not been elucidated yet.

#### Liquid hot water (LHW)

Liquid hot water is another physico-chemical pretreatment that employs water at elevated temperature and high pressure so that water maintains its liquid form (Mok and Antal 1992; Rogalinski et al. 2008). Temperature adopted changes from 150 to 240 °C and times from several minutes up to few hours with temperature dominating the type of sugar (pentose and hexose) and time influencing sugar yields (Yu et al. 2010). For example, Yu et al. (2010) showed that 180 °C and 30 min are the most cost-effective pretreatment conditions for rice straw bioconversion into glucose.

LHW has been shown to be an efficient method to remove up to 80 % of the hemicellulose in for pretreatment of different kinds of lignocellulosic materials including sugar cane bagasse (Laser et al. 2002), corn stover (Mosier et al. 2005), wheat straw (Perez et al. 2008), and sunflower stalks (Monlau et al. 2012).

This process is able to solubilize up to 80 % of the hemicellulose and separate it from cellulose and lignin residues. In particular, water penetrates into the biomass cell structure solubilizing the hemicellulose and promoting at the same time the alteration of the lignocellulosic matrix, producing more accessible cellulose (Kim et al. 2009). In fact, after LHW pretreatment, two products are formed: the solubilized hemicellulose and a solid fraction that includes lignin and cellulose that must be isolated from the lignin fraction (Perez et al. 2008). The solid fractions are more susceptible to enzymatic hydrolysis (Zeng et al. 2007). Therefore, it is important to lessen the cellulose solubilization, while keeping hemicellulose solubilization high.

LHW shows high sugar recovery after enzymatic hydrolysis (80 % xylose and arabinose and 94 % glucose) (Dien et al. 2006) and shows the potential to improve sugar recovery and cellulose digestibility, producing less inhibitor compounds, compared with catalyzed SE or acid catalyst. Actually, during LHW pretreatment, the cleavage of O-acetyl and uronic acid produces organic acids (such as acetic acid) that help to increase the hydrolysis of polysaccharides into soluble monosaccharides that are then partially degraded to other inhibitors (furfural and HMF) (Perez et al. 2007). In addition, at high temperatures, water acts like an acid. Therefore, in order to prevent the formation of inhibitors, the pH should be kept between 4 and 7. For example, Laser et al. (2002) modifying operating parameters (such as temperature and reaction time) while keeping the fixed pH during LWH the hydrothermal pretreatment of corn stover, they obtained maximum hemicellulose solubilization and 90 % of the cellulose conversion at 190° and 15 min and little formation of inhibitors. LHW pretreatment has been already implemented and optimized on pilot scale at DONG Energy facility in Denmark after in-deep economic feasibility studies. In fact, Tao et al. (2011) compared different pretreatment processes to convert switchgrass to monomeric sugars for a techno-economic analysis. In this study, all processing conditions such as residence time, solid loading, and temperature are considered, and the possible chemical recovery step has been considered. Considering continuous process configuration in a large-scale plant with theoretical ethanol yield of 87.7 million gallons per year, LHW required the lowest capital investment (20 million dollars) between all methods investigated.

#### Advantages and drawbacks

This pretreatment is attractive because it does not require addition of chemicals or catalysts, and, unlike SE, it does not require rapid decompression or expansion and because biomass size reduction which is a highly cost operation especially when applied on industrial scale is not needed prior to the process (Taherzadeh and Karimi 2008). Moreover, pretreatment reactor costs are low and it produces less inhibitor compounds than other methods, while keeping high sugar yields. The main drawbacks of this process are the high energy demand compared to SE due to the high pressure and the large amount of water required by the system. However, this process is attractive for large-scale operations because expensive reactor systems resistant to corrosive chemicals are not necessary (Petersen et al. 2009). In fact, LHW pretreatment has been implemented and optimized on pilot scale at DONG Energy facility in Denmark.

#### Ammonia fiber explosion (AFEX)

In the ammonia fiber explosion (AFEX) process, biomasses are exposed to 1–2 kg liquid anhydrous ammonia per kg of dried biomass, under high pressure (17–20 bar), and unexcessive temperature (60–100 °C) from short (5–10 min) to long residence times (30 min), and then rapidly undergoes a pressure release, as it occurs in SE pretreatment. The temperatures, which are lower than that of SE, decrease the energy and economic demand of the entire process (Mes-Hartree et al. 1988).

The rapid expansion of ammonia causes physical breaking down of the lignin-polysaccharide cross-links, hemicellulose degradation to oligomeric sugars, successively deacetylated, and partial decrystallization of cellulose while lignin remains unaffected (Laureano-Perez et al. 2005; Wyman et al. 2005a). Therefore, the structure of native materials changes resulting in higher digestibility in enzymatic hydrolysis step and higher sugar yields. However, hemicellulose is not significantly solubilized in the AFEX pretreatment compared to other methods such as acid pretreatment or catalyzed SE (Mes-Hartree et al. 1988; Vlasenko et al. 1997); thus, also hemicellulase is required in enzymatic hydrolysis step. Moreover, AFEX process provides only a solid fraction differently from the other pretreatments that produce a slurry containing a liquid and solid fraction, (Mosier et al. 2005). Parameters such as temperature, moisture content, ammonia loading, and residence time can be modified in order to increase monomeric sugar yields. In fact, when optimal conditions are employed, although there is little lignin removal in comparison with other methods, it is possible to obtain 90 % conversion of cellulose and hemicellulose (Wyman et al. 2005b). However, the optimum process conditions depend on the feedstock. A wide variety of lignocellulosic biomasses, such as corn stover (Balan et al. 2009), bagasse (Holtzapple et al. 1991), municipal solid waste (Holtzapple et al. 1992), and rice straw (Vlasenko et al. 1997) have been pretreated with this method. However, it has been shown that AFEX is more efficient on agricultural residues while it is not effective for biomass with high content of lignin such as hardwood (Holtzapple et al. 1991).

Ammonia recycle percolation (ARP) is another process utilizing ammonia in which the materials are pretreated with 10–15 wt% of aqueous ammonia in a flow-through reactor, with temperature varying between 150 and 200 °C, percolation rate of 5 mL/min and residence time of 14 min, and then the ammonia was recovered (Kim et al. 2008). ARP can solubilize almost half hemicellulose but it retains up to 90 % of cellulose, in particular a short cellulose chain with high glucan content (Yang and Wyman 2008). This method retains all the advantages of AFEX with the only difference in using aqueous ammonia and not liquid ammonia.

A more recent methodology that employs the use of ammonia, called extractive ammonia (EA), convert native crystalline cellulose I_β_ to another allomorph high digestible, cellulose III_I_, resulting in higher enzymatic hydrolysis rate. Unlike AFEX, this novel process can extract up to 45 % lignin without modifying lignin functionalities. In this way, lignin can be valorised and used as a renewable chemical feedstock for development of other value-added products in biorefinery application (da Costa et al. 2016).

Finally, it is clear that all these technologies must evolve, in future years, toward ammonia loading reduction and development of low-cost ammonia recycling step in order to respect environment, reduce overall cost of the process, and make this method more green for biorefinery industry development. In fact, respect to economic feasibility of the process, Tao et al. (2011) studied the economic feasibility of a commercial-scale IL pretreatment for an 87.7 million gal/year switchgrass as feedstock for the base case. They assessed that total capital investment for this technology is very high, 348 million dollars, compared to other method, such as LHW. The AFEX pretreatment processes in fact, have significant equipment requirements related to recovery of chemicals, which is an important contribution to the overall costs. However, the design of these pretreatment chemical recovery and recycle systems is preliminary. So, further techno-economic study and research to develop new configuration system for chemical recovery are necessary and may lead to lower overall pretreatment system capital costs.

#### Advantages and drawbacks

The major advantages of this pretreatment include: high reducing sugar yields (up to 80–90 %), low sugar degradations, and no generation of inhibitory compounds, even though some phenolic fragments of lignin and other cell wall extractives may remain (Bradshaw et al. 2007). A great limitation in using AFEX is the high volumes of adopted ammonia that increase process costs. However, recycling of ammonia during the process can decrease the operating costs and environmental pollution (Eggeman and Elander 2005).

#### Supercritical CO_2_ explosion

Supercritical CO_2_ explosion is a pretreatment method that uses, as a “green solvent”, supercritical fluid that is a material that exists at a temperature and pressure above its critical point where gas and liquid phase are not distinguished. In fact, the replacement of common chemicals and solvents by safer ones is becoming necessary in order to help the development of low environmental impact technologies (Cherubini 2010; Morais and Bogel-Lukasik 2013). Thus, supercritical fluids can be employed to disrupt the crystalline structure of lignocellulose and efficiently remove lignin, enhancing cellulose digestibility (McHardy and Sawan 1998). Among them, supercritical CO_2_ (SC-CO_2_) shows excellent potentiality for lignocellulosic biomass pretreatment. Zheng et al. (1995) were the first who used CO_2_ explosion as pretreatment method for commercial cellulosic materials (Avicel) in 1995, and they used this method again in 1998 to treat recycled paper mix and sugarcane bagasse. They showed up to 75 % improvements in glucose yields compared to untreated biomass (Zheng et al. 1998). Nowadays, the number of articles related to the use of CO_2_ under sub- or supercritical conditions, as a green solvent for biomass pretreatment in a biorefinery concept, is increasing (Morais et al. 2014; da Silva et al. 2014; Schacht et al. 2008) and it is expected that it is going to grow in the near future. The energy demand needed to bring CO_2_ to its supercritical point is lower than those required for other solvents, thanks to its low critical temperature (31°) and pressure (73.8 bar) (Sheldon 2005).

CO_2_ molecules are capable of penetrating into small pores of lignocellulosic materials, as water and ammonia; so, SC-CO_2_ explosion process is basically the same as AFEX and SE pretreatments. In contrast with SE, SC-CO_2_ explosion shows lower reducing sugar yields and does not cause degradation of sugars, due to lower temperature adopted, and does not produce inhibitory compounds; while, in comparison with AFEX, it is less costly (Zheng et al. 1995).

The “explosion” of CO_2_ helps the alteration of biomass structure, decreasing the degree of crystallinity and increasing the permeability, the accessibility, and surface area of cellulose (Zheng et al. 1998). This results in higher amounts of reducing sugars after enzymatic hydrolysis unlike untreated biomass. Parameters influencing SC-CO_2_ explosion process are temperature, residence pressure, treatment time, moisture content, and CO_2_/biomass ratio. For example, temperature is a relevant factor because the experiments can be carried out at either sub- or supercritical temperature. Kim and Hong (2001) pretreated aspen wood and yellow pine with CO_2_ explosion under supercritical conditions. They showed that both pretreated biomasses produce higher amounts of reducing sugars (from 14.5 and 12.8 % to 84.7 and 27.3 %) after enzymatic hydrolysis in comparison with native materials. In another work, Zhang and Wu (2014, 2015) demonstrated that SC-CO_2_ explosion pretreatment of eucalyptus chips and sugarcane bagasse has a positive influence on enzymatic hydrolysis, also under subcritical conditions. They reported glucose yields of 92.2 and 93 % that are higher than for untreated eucalyptus feedstock and sugarcane bagasse, respectively. Pressure is another important factor that influences the process. Alinia and co-workers (Alinia et al. 2010) examined the effect of this parameter on wheat straw pretreatment. They concluded that changing the pressure from 80 to 120 bar, there is an increase of sugar yields, but increasing the pressure above 120 bar did not significantly change sugar yields.

Another interesting technology is the addition of co-solvents such as ethanol–water or acetic acid–water to supercritical CO_2_. This process can improve delignification process for several reasons as follows: CO_2_ enhances capacity of ethanol to dissolve lignin; dissolution of CO_2_ in water causes carbonic acid formation that increases enzymatic hydrolysis rate; and water is a polar solvent that can break the bonds between hemicellulose and lignin (Pasquini et al. 2005). All these advantages produce a pretreatment that remove the potential cellulase inhibitors (Huisheng et al. 2013).

After these considerations, SC-CO_2_ process seems to be too expensive for the development of an industrial scale plant because of the high cost associated to the high quantity of CO_2_ required. However, in literature does not exist a techno-economic analysis of this method, so further studies are necessary prior to design a pilot plant reactor.

#### Advantages and drawbacks

SC-CO_2_ explosion pretreatment is environmentally friendly since CO_2_ is considered as a green solvent because it is non-toxic (unlike ammonia) and non-flammable (Sheldon 2005; Gu et al. 2013). Moreover, CO_2_ can be recycled through photosynthesis, therefore its release in the atmosphere after pretreatment does not mean necessarily an increase of CO_2_ emission. Furthermore, this process does not create waste products and does not require further CO_2_ recovery because CO_2_ can be easily removed by depressurization (Schacht et al. 2008). In comparison with physico-chemical pretreatment, this method shows lower reducing sugar yields, does not cause degradation of sugars, does not produce inhibitory compounds, and is less costly. Apart from all the aforementioned advantages, the supercritical CO_2_ process seems to be too expensive for industrial application. Thus, improvements are necessary to carry out the process on a large scale.

### Chemical pretreatments

Among the pretreatment categories, chemical pretreatment deserved great scientific interest in the last years. To this group belong the pretreatments that use chemicals to disrupt the biomass structure. However, in the green biorefinery, it is necessary to develop technologies that aim at eliminating the use and/or generation of chemicals. For this reason, this review is focused only on organosolv, ozonolysis and ionic liquids (ILs) pretreatments because organic solvents, ozone and ILs are considered “green” solvents since they are non-toxic and do not produce hazardous wastes.

#### Organosolv pretreatment

Organosolv pretreatment is a promising chemical pretreatment method that employs organic solvents such as methanol, ethanol, ethylene glycol, or acetone or mixture of organic solvents and water, to solubilize and isolate lignin from lignocellulosic biomasses breaking internal lignin bonds and also lignin-hemicellulose bonds (Ichwan and Son 2011; Pan et al. 2006). In this method, materials are mixed with organic solvents and water and heated, at temperature of 150–200° depending on the type of materials, to dissolve lignin and hemicellulose, leaving cellulose in the solid residue. Extraction of lignin during pretreatment step leads to an increased cellulose accessibility to enzymes during saccharification step (Zhao et al. 2009a). Moreover, from the environmental point of view, extraction of lignin as a by-product decreases the problem with waste treatment. In some cases, it is possible to add an organic or inorganic catalyst (HCl, H_2_SO_4_ oxalic, or salicylic acid) in order to decrease operating temperature and increase sugar yields (Zhao et al. 2009a). However, the presence of catalyst can degrade monosaccharides into furfural and 5-hydroxymethyl furfural (HMF) that are inhibitory compounds for enzymatic hydrolysis. For example, Park et al. (2010) evaluated the effect of three different catalysts (H_2_SO_4_, MgCl_2_, and NaOH) on pine pretreatment with ethanol and different temperatures. H_2_SO_4_ showed the best efficiency even at low temperatures (55–60 % digestibility); MgCl_2_ was efficient (60 % digestibility), but required high energy input (digestibility increases as temperature and residence time increases); the pretreatment with NaOH had no effect on cellulose digestibility (10 % digestibility).

Organosolv process can be also combined with other pretreatment such as dilute acid hydrolysis to separate hemicellulose and lignin in a two-stage fractionation process. This two steps process seems to produce a material enriched in cellulose, increase cellulose digestibility and yields of pure lignin, and avoid losses of sugars from hemicellulose.

For example, Mesa et al. (2011) used organosolv ethanol pretreatment on sugarcane bagasse previously pretreated with dilute acid hydrolysis with adding of NaOH under different operational conditions (pretreatment time, temperature, and ethanol concentration) in order to evaluate and maximize sugar yields. They showed that optimum conditions consisted in using 30 % (*v*/*v*) ethanol at 195 °C.

The solvents itself can be inhibitor for saccharification and fermentation step, therefore, removal of them is necessary. Solvents can be removed using extraction and separation methods, such as evaporation. In this way, they should be recycled resulting in reducing operational costs and environmental impact. Moreover, it is important to consider the high commercial price of solvents for the development of industrial scale plant. Therefore, in organosolv process, low molecular weight alcohols, such as ethanol that are less toxic than others, are preferred.

#### Advantages and drawbacks

The main advantage of organosolv method, in comparison to other methods, is the recovery of an almost pure lignin as a by-product (Zhao et al. 2009a). In fact, lignin can be separated from the solvent and used to produce added-value chemicals based on lignin (Pan et al. 2005). Moreover, separation of lignin before enzymatic hydrolysis can enhance cellulose digestibility resulting in a lower enzymes quantity, and consequently, reduction of the costs. Moreover, ethanol as solvent produces low hemicellulose degradation products and promotes high solid recovery and protection of the cellulose fraction. The main disadvantages of this process include necessity of recycling step for solvents, in order to decrease the overall costs and the inhibitory effect of solvents on enzymatic and fermentation steps, and flammability of the solvents (Sun and Chen 2008). This is an important economic and safety aspect because additional equipment, that increases costs, is necessary in order to avoid possible fires and explosions.

#### Ozonolysis pretreatment

Ozone is an effective oxidant which can be employed in ozonolysis pretreatment of lignocellulosic biomasses.

Ozone could attack aromatic rings structure resulting in damage of lignin, by damaging aromatic rings structure and could release other soluble compounds such as acids (acetic, formic, caproic, levulinic, *p*-hydroxybenzoic, vanillic, and malonic) and aldehydes (*p*-hydroxybenzaldehyde, vanillin, and hydroquinone) (Morrison and Akin 1990). The degradation occurring in this method was essentially limited to lignin and partially to hemicellulose while cellulose was very little altered. This results in an increased cellulose biodegradability and sugars yield after enzymatic hydrolysis step. Garcia-Cubero et al. (2009) obtained up to 88.6 and 57 % sugar recovery after pretreatment of wheat and rye straw, respect to 29 and 16 % of untreated samples.

Several agricultural residues, such as wheat straw (Garcia-Cubero et al. 2009), bagasse (Travaini et al. 2013), peanut and poplar sawdust (Vidal and Molinier 1988), and olive mill waste (Benitez et al. 1997), have been pretreated with this method. However, several researchers have shown that ozonolysis alone is ineffective to remove lignin and increase yields of reducing sugars, while, combination of this method with others has exhibited promising results. For example, de Barros et al. (2013) studied the effect of ozonolysis in combination with wet disk milling (WDM) on sugarcane bagasse and straw to improve sugar yields after enzymatic saccharification. Under optimized conditions, they obtain 89.7 % of glucose and 48.8 % of xylose yield from sugarcane bagasse and 92.4 and 52.3 % from straw. After these considerations, ozonolysis method seems to be too expensive for the development of an industrial scale plant because of the high cost associated to the high quantity of ozone required. However, does not already exist an economic analysis, so further studies are necessary.

#### Advantages and drawbacks

Among the main advantages of this method are moderate reaction conditions (such as room temperature and atmospheric pressure) and the little generation of toxic residues and inhibitory compounds that can influence subsequent steps, efficient degradation of lignin, and lower environmental pollution. Despite of some interesting results and the many pros of this pretreatment, such as efficient degradation of lignin, moderate reaction conditions, lack of degradation by-products and toxic residues, and lower environmental pollution, an important drawback is the high costs of needed ozone, resulting in a process economically and industrially unfeasible (Sun and Cheng 2002).

#### Ionic liquids (ILs) pretreatment

Ionic liquids (ILs) are termed “green solvents” since no toxic chemicals are formed and since nearly 100 % of solvents can be recovered (Heinze et al. 2005). Thus, ILs pretreatment can be considered as a green method. ILs are particular salts that can exist in liquid form at temperatures lower than 100 °C and also at room temperatures, made up of big organic cations and smaller inorganic anions (Marsch et al. 2004). Their properties, which are different from those of other common organic solvents, can change by varying degree of anion charge delocalization and the length and the symmetry of alkyl constituents of the cations. Thanks to these properties, such as low toxicity, low hydrophobicity, high viscosity, low vapor pressure, thermal stability, enhanced electrochemical stability, and non-flammable properties, their use have low energy imputs and potentially minimal environmental impact. Therefore, in the last years, the use of ILs to dissolve many type of lignocellulosic biomasses, such as corn stover (Cao et al. 2010), bagasse (Qiu et al. 2012), wheat straw (Li et al. 2009), and poplar (Lucas et al. 2010), has received more attention. In particular, ILs dissolution of biomass occurs at ambient pressures and temperatures of 90° to 130° from 1 h to 24 h (Zhu et al. 2006).

Many types of Ils have been utilized to date, but imidazolium-based Ils have received the most attention. However, in the recent years, Ils containing cholinium cations and amino acids anions, known as bionic liquid, were shown to be efficiently to pretreat different type of lignocellulosic biomass by removing lignin. These Ils are considered greener and more economic solvents than imidazolium-based one because they derived from natural and renewable starting materials (Liu et al. 2012; Hou et al. 2012). Moreover, comparing sugar yields after pretreatment of switchgrass with amino acid-based and imidazolium-based Ils, the former produce higher glucose and xylose yields (96.5 and 95.7 % compared to 95.8 and 93.3 %) (Sun et al. 2014).

Sun et al. (2011) found out that the source of cellulose (feedstocks), cations, anions, temperature, and time employed in this method are the principal factors affecting biomass dissolution. For example, cellulose can be efficiently dissolved in ILs containing anions, such as chloride, acetate, or amino acids, because these anions form strong hydrogen bonds with sugars’ hydroxyl protons (Zhao et al. 2009b; Zavrel et al. 2009). On the other side, the π-π interactions of the cations with lignin help lignin solubilization (Shill et al. 2011). As a result, the degradation of the complex network of interactions between cellulose, hemicellulose and lignin is observed, with reduced degradation products. Cellulose, recovered with anti-solvents such as ethanol, acetone, or water, seems to have the same number of glycosyl residues (known as degree of polymerization) as the untreated cellulose, but decreased crystallinity and increased porosity. As a result, it becomes more susceptible to degradation by cellulase with an increased yield of glucose (Zhu 2008; Wang et al. 2012). Also, lignin can be isolated and recovered after ILs pretreatment; it contained the highest molecular mass molecules, did not contain new condensed structures, and was depolymerized. Thus, it may be more amenable to be used as a renewable chemical feedstock for other applications resulting in a decreased biorefinery costs (Sathitsuksanoh et al. 2014).

Further researches are needed to apply ILs pretreatment at industrial scale, in a biorefinery concept. First of all, an economic study is necessary. Baral and Shah (2016) assessed the techno-economic feasibility of a commercial-scale IL pretreatment for a 113 million liter/year (30 million gal/year) plant using corn stover, poplar, and switchgrass as feedstock for the base case. They identified ionic liquid recovery, ionic liquid cost, and heat recovery as the most sensitive process parameters for techno-economic study. They evaluated total capital investment and its different components, such as total fixed capital, working capital, and startup cost, and estimated that this total investment costs were 873, 941 and 1002 $/t total sugars, for corn stover, poplar, and switchgrass, respectively.

Currently, another solvent, *N-methylmorpholine N-oxide* (NMMO), is used commercially to generate cellulose fibers, in the eco-friendly Lyocell process, with direct dissolution of the cellulose not requiring chemicals addition (Perepelkin 2007). This solvent keeps all the advantages of ILs: it is useful for dissolution of cellulose and to modify the crystallinity of cellulose from a variety of materials breaking the hydrogen bond network of the cellulose, it is non-toxic, and because of its low vapor pressure, it can be recovered up to 100 % (Kuo and Lee 2009a; Kuo and Lee 2009b; Shafiei et al. 2010; Rosenau et al. 2001). Moreover, another important advantage of this solvent is the easy recovery of cellulose, by addition of water as an anti-solvent. Finally, compared to the other pretreatment methods, NMMO does not change the composition of the materials because it does not remove hemicelluloses.

#### Adavantages and drawbacks

There are many disadvantages in using this pretreatment: great amounts of expensive ILs are needed, hemicellulose and lignin must be recovered after cellulose extraction, and solutions become viscous and difficult to handle during the process. Fu and Mazza (2011) proposed the use of water-mixtures of ILs in order to avoid this problem. Moreover, an important limitation in using this pretreatment is that, in some studies, ILs showed negative effect on cellulase activity because it leads to the unfolding and irreversible inactivation of the enzyme (Turner et al. 2003; Yang et al. 2010). Thus, ILs residues removal is a necessary step before enzymatic hydrolysis in order to preserve cellulase activity and increase the yield of sugars, even though this step has not been fully developed and increases the overall cost of the process.

Despite of these drawbacks, ILs pretreatment shows great potential because it is considered more environmentally friendly than other chemical pretreatment methods (Hayes 2009) and is comparable in sugar yields. For example, Xu and co-workers (Xu et al. 2012) achieved, after enzymatic hydrolysis of corn stover pretreated with [C_2_mim] OAc, monosaccharides yields of 84.9 and 64.8 % (glucose and xylose, respectively), comparable to the yields of AFEX pretreatment (82.0, 72.2, and 78.4 %, respectively) (Li et al. 2011).

### Biological pretreatments

Biological pretreatment is getting great relevance because it is an effective, safe, and environmentally friendly approach (Wan and Li 2012; Sindhu et al. 2016). The abovementioned pretreatment methods in fact require expensive equipment, high energy inputs, and sometimes the use of harsh chemicals resulting in environmental pollution. Biological pretreatment instead, employs the use of microorganisms, such as fungi (Eriksson et al. 1990), bacteria (Varm et al. 1994), and actinomycetes, that synthetize cellulolytic, hemicellulolytic, and ligninolytic systems to degrade lignin, cellulose, and hemicellulose.

Biological pretreatment that employs the use of actinomycetes degrades lignin into low molecular weight fragments. For example, Saritha and Arora (2012) pretreated softwood and hardwood with *Streptomyces griseus* showing higher lignin loss (10.5 and 23.5 % respectively) compared to untreated biomass. However, research is mainly focused on the use of wood rot fungi (white-, brown- and soft-rot fungi), and in particular on evaluating which genera and species of fungi delignify biomass without degrading cellulose (Sánchez 2009; Anderson and Akin 2008). Fungal pretreatment is carried out via a solid-state fermentation (SSF) process depending on process parameters such as temperature, moisture content, and aeration (Wan and Li 2012). Therefore, different types of bioreactors, with different parameters, have been developed because a single method cannot be universal for all types of biomass. Among wood-rot fungi, white-rot were shown to be the most effective and common used in the pretreatment of many type of biomasses such as wheat straw (Pinto et al. 2012), rice straw (Taniguchi et al. 2005), softwood (Lee et al. 2007), and Bermuda grass (Akin et al. 1995). Many types of white-rot fungi, such as *Phanerochaete chrysosporium*, *Clostridium butyricum*, *Trichoderma viride*, *Pycnoporus cinnarbarinus*, *Dichomitus squalens*, *Phlebia radiate*, *Trametes versicolor*, *Aspergillus oryza*, basidiomycete Euc-1 and *Pleurotus ostreaus*, have shown good delignification efficacy (Shi et al. 2008). For example, wheat straw was pretreated by two different white-rot fungi, Euc-1 and *Irpex lacteus*, in order to test the improvement of cellulose accessibility (Dias et al. 2010). Results of this study indicated that the cellulose accessibility increased from 2.7, in the untreated biomasses, to 5.9 after the pretreatment by the basidiomycetes Euc-1, thus proving its highly lignin biodegradation capability. However, among the known species of white-rot fungi, the highest efficiency in lignin degradation belongs to *P. chrysosporium* due to its high growth rate. In fact, Shi et al. (2009) showed that 19.38 and 35.53 % lignin degradation of cotton stalks was obtained after biological pretreatment with this microorganism in solid-state cultivation (SSC).

Other microorganisms used in biological pretreatment are brown-rot fungi (*Tyromyces balsemeus*, *Poria placenta*, *Lentinus lepidius*, *Fomitopsis pinicola*) that can degrade cellulose and hemicellulose with slight modification of lignin structure (Rasmussen et al. 2010). Endo-cellulases, exo-cellulases, cellobiohydrolase, and β-glucosidases cause cellulose degradation (Baldrian and Valášková 2008); while, action of the action of other enzymes such as endo-xylanases, endo-α-L-arabinase, β-galactosidase, and β-glucosidases induces hemicellulose degradation (Shallom and Shoham 2003). These microorganisms are capable of completely degrading lignin to CO_2_ and H_2_O due to the action of lignin-degrading enzymes such as laccases and lignin and manganese peroxidases that are regulated by carbon and nitrogen sources (Shi et al. 2009; Howard et al. 2004). In fact, these enzymes are produced by many white-rot fungi during secondary metabolism, in response to carbon or nitrogen limitation (Boominathan and Reddy 1992). Lignin peroxidases are hemeproteins, with Fe^3+^ in a porphyrin ring, involving in the oxidative cleavage of phenolic and non-phenolic aromatic substrates (Wong 2009). Manganese peroxidase is an enzyme secreted to help lignin degradation by the oxidization of Mn^2+^ into Mn^3+^ (Hofrichter 2002). Laccases are blue, multicopper oxidases that catalyze the oxidation of phenolic, and other electron-rich substrate (Tong et al. 2007).

These enzymes have been adopted for many biotechnological processes in order to convert lignocellulosic biomasses into value-added products through a “green” process, reducing energy demand, and environmental pollution. In fact, the main pros of this method include moderate conditions, higher delignification rate, no nutrient addition, and no sugar consumption (Ibarra et al. 2006; Vivekanand et al. 2008).

Another type of biological pretreatment is bacterial pretreatment, because in the recent years, bacteria showed their ability to degrade lignin through laccases. In particular, laccases from *Azospirillum lipoferum* (Diamantidis et al. 2000), *Bacillus subtilis* (Martins et al. 2002), and *Streptomyces lavendulae* (Suzuki et al. 2003) have been characterized.

Despite fungal laccases have higher redox potential than bacterial laccases, recently the research was aimed to develop pretreatment method, for bioconversion of lignocellulosic biomasses into a wide range of bioproducts, through the microbial digestion (Liang et al. 2014; Tang et al. 2015). In fact, bacteria show lower level of (hemi)-cellulolytic activity production, that reduces the loss of cellulose in comparison with fungal pretreatment. For example, Liang et al. (2014), use undefined mixed cultures in order to convert potato peel waste (PPW) into lactic acid, varying different conditions during pretreatment and enzymatic hydrolysis, such as temperature, pH, nutrients, and solids loading.

Although, huge economic information about other pretreatment methods such as SE, AFEX, and LHW, has been reported, few references exist on the economic aspects of biological pretreatment (Saritha and Arora 2012). Therefore, there is an urgent need for research for the development of a realist process at pilot plant or larger scale. For example, using metabolite of a microorganisms present in nature is an eco-friendly and a cheaper strategy for enhancing enzymatic saccharification rate, since no chemicals were used in this process, there is no need for recycling of chemicals and no release of toxic compounds to environment. Another way to decrease costs of the process is to conduct pretreatment and saccharification in the same vessel, resulting in a decreased energy consumption and easier process. Moreover, further researches are necessary to isolate and purify new cheaper enzymes (Sindhu et al. 2016).

#### Advantages and drawbacks

It is clear that this method can be considered as a “green” method. In fact, it is cost-effective and easy to operate, it requires low energy inputs, no chemical addition and milder conditions, resulting in few inhibitors generation, and it does not cause environmental pollution. However, this method is affected by many disadvantages. In general, the main drawbacks are the low hydrolysis rate obtained, the necessity of a large sterile area which should been maintained during all process, the slow growth rate of the fungi that limits on-farm scale application, and protracts process time and the need for monitoring this microorganisms growth.

## Conclusions

Lignocellulosic biomasses are interesting alternative to fossil-based resources and are the prime sources utilized in green biorefineries for the production of bioenergy and other value-added bioproducts. However, the use of sustainable resources is not enough to protect the environment. In fact, it is important that the methods used in all steps of biomass conversion minimize environmental pollution and produce sustainable bioproducts, without producing a lot of wastes and increasing the costs of the process. Among all steps required in lignocellulose bioconversion, the pretreatment is crucial for enhancing cellulose accessibility to the enzymes. Several pros have been reported for most of the pretreatment methods, making them interesting for scale-up applications; however, one method could not be the universal choice for all types of feedstock and there is still little literature about “green” techniques. In the last years, the integration of green chemistry in biorefineries is offering new protocols when developing pretreatment processes. However, further research is required to make the products more competitive than those obtained from petroleum industries.

For example, among physico-chemical pretreatment, extrusion seems one of the most environmentally friendly because it is chemical-free or it uses non-toxic chemicals and employs moderate conditions and cost-effective equipment. However, until now there are no articles on fermentation results after extrusion. Therefore, further studies about fermentation are mandatory in order to develop this method in biorefineries. Moreover, less sugar recovery is observed using extrusion method alone, hence, a combination of two or more pretreatments have to be deepened.

Hydrothermal methods, including LHW and SE, have been widely tested throughout the years because, despite the disadvantage of using harsh conditions (such as high temperatures and pressures), they give high sugar yields, not requiring the addition of chemicals or require the addition of non-toxic chemicals that can be recycled, resulting in a reduced environmental pollution.

SC-CO_2_ pretreatment also shows excellent potentiality for pretreatment of a wide range of lignocellulosic biomasses, using different operating conditions. However, further researches are mandatory to investigate the possibility of CO_2_ recycling without increasing overall process costs.

Instead, among chemical methods, ILs pretreatment shows great potential as “green” method. However, as well as for SC-CO_2_ pretreatment, further researches are necessary to apply ILs at industrial scale because of high initial cost of solvents. One solution to reducing the costs can be the development of methods that use chemicals (such as amino acids) that make ILs biodegradable and easily recycled.

Regarding biological pretreatment, this method is limited by some drawbacks, such as the identification of optimal culture conditions, in addition to the high variability of the process, due to different microorganism growth rate and percentage of humidity. Therefore, future investigations for the optimization of this method must be aimed to improve enzyme and microorganism performances, with a focus on to the economic feasibility for the industrial scale-up.
